# The Paradox of Digital Health: Why Middle-Aged Adults Outperform Young Adults in Health Management Utilization via Technology

**DOI:** 10.3390/healthcare12222261

**Published:** 2024-11-13

**Authors:** Seo-Ha Jeong, Yeon Gyo Nam

**Affiliations:** Department of Physiotherapy, College of Health Science, Sun Moon University, Asan 31460, Republic of Korea

**Keywords:** digital health, health surveys, digital divide, population health management, health promotion

## Abstract

**Background/Objectives:** Globally, life expectancy has been increasing with South Korea focusing on improving health to enhance quality of life. The COVID-19 pandemic further emphasized the need for digital transformation in healthcare, accelerating digital health adoption. This study explores the digital divide between ‘Digital Natives (20–39 Y)’ and ‘Digital Immigrants (40–69 Y)’, focusing on digital device usage and confidence. **Methods:** This study utilized national survey data from the Digital Health Literacy Survey Results and Policy Implications, focusing on differences in digital device use and confidence between young adults (20–39 Y) and middle-aged adults (40–69 Y). The participants comprised 1000 adults aged 20 to 69 in the Republic of Korea. Respondents were queried about their use of digital health tools, such as wearable devices and mobile apps. Confidence in using digital systems and managing health via digital tools was assessed using a five-point Likert scale. **Results:** The findings indicated that while young adults have lower rates of using digital devices for healthcare, they exhibit higher confidence in using such devices. In contrast, middle-aged adults, despite having lower confidence, report higher usage of digital devices for healthcare purposes. **Conclusions:** This study explored differences in digital confidence and healthcare usage between age groups and aimed to propose effective health management strategies based on digital accessibility.

## 1. Introduction

According to research, globally, life expectancy has been on the rise over the past few decades [1,2]. Among the countries of the world, South Korea has made significant strides in life expectancy in recent years. As of the latest data, life expectancy at birth is approximately 85.6 years [1].

Is increasing life expectancy simply beneficial? People with illnesses or not being healthy show increased levels of depressed mood and decreased eudemonic wellbeing. Health is the leading cause of well-being [3]. Easterlin 2003 research also emphasized that declines in health have a sustained negative impact on happiness [4]. Since good health is the basis of a high quality of life, people can live longer and healthier lives by managing health habits and delaying the process of aging [2,5]. Nature Index, 2023 highlighted that research institutions globally are increasingly focusing on health-related studies, which reflects a growing awareness and prioritization of health issues across multiple disciplines. Especially in the United States, health-science research is a major focus of federal spending [6]. The rationale behind this is that knowledge of health creates the precondition for an individual’s behavioral change and significantly impacts lifestyle habits [5].

Furthermore, awareness and behaviors focused on maintaining health have increased during the COVID-19 pandemic [7]. Throughout this period, individuals actively practised or sought out relevant healthcare behaviors in their daily lives, demonstrating a global response to the pandemic [8,9]. According to the Hong 2022, 89 percent of Koreans are aware of ‘the importance of preventive health care’, and 51 percent of Koreans have become more proactive in practising self-care to maintain their health. After the COVID-19, 30 percent reported being more concerned about “acquiring information on health and disease prevention”. This is because sufficient knowledge and information are crucial for maintaining a healthy life [10].

The COVID-19 pandemic also highlighted the limitations of analog methods in healthcare systems. To overcome this crisis, the digital and technological revolution in healthcare has transformed the global landscape [11]. As a result, the pandemic led to a rapid increase in the adoption of digital health technologies [12].

Several previous studies have examined the impact of digital health technologies on health outcomes [13,14,15,16,17,18,19,20]. Digital health is defined as the application of information communication technology to support health through electronic and mobile health solutions, including the use of big data, computational genomics, and artificial intelligence [21]. Digital health has the potential to improve population health by increasing access to medical services [21,22,23,24,25]. The scope of digital health includes interventions such as mobile applications, wearable devices, social media, telehealth, telemedicine, and interactive websites [15,17,18,26,27,28,29].

“Digital Natives” and “Digital Immigrants” are terms coined by Prensky in 2001 to describe the current tech-savvy generation. ‘Digital Natives’ refers to individuals who have been exposed to digital technology from a young age, integrating it into their daily lives from the beginning. On the other hand, ‘Digital Immigrants’ refers to individuals who were not born into the digital world but have adopted technology later in life, often having to adapt and learn new digital skills as adults [30]. One instance of the digital divide is digital confidence. According to Hoffmann 2014, younger learners exhibit a high level of digital confidence, whereas digital immigrants, who were more likely to be older boomers, demonstrate significantly lower levels of confidence [31]. In this context, the digital divide between ‘Digital Natives’ and ‘Digital Immigrants’ is of great interest to managers attempting to cope with escalating uncertainty and volatility in today’s market [32]. Furthermore, this divide has significant social, political, cultural, and economic implications [33].

Existing studies have covered the overall content of the survey [34]. The Korea Information Society Agency conducted a Digital Information Gap Survey, analyzing digital information levels among various demographic groups in 2022 South Korea. The survey revealed that the lowest levels were observed in the elderly population (68.6%), followed by farmers and fishermen (77.3%), individuals with disabilities (81.3%), and low-income individuals (95.1%). The elderly participants were primarily in their 50s, 60s, and 70s. Given this context, it was inferred that disparities in digital information levels may also exist among other age groups. Consequently, this study has chosen to focus on the variable of ‘age’ as its central theme [35]. The criteria for categorizing age groups were flexibly adapted from Erikson’s 1994 theory of psychosocial development [36] and the report from the Ministry of Employment and labor (MOEL. 2017) [37]. Erikson’s theory posits that individuals’ egos and personalities develop through eight stages, each presenting new decisions and turning points throughout life. Adulthood stages were delineated into three stages: (1) Intimacy vs. Isolation (early and emerging adulthood, 20–40 years), (2) Generativity vs. Stagnation (adulthood, 40–65 years), and (3) Ego Integrity vs. Despair (maturity, 65+). Young-adults were defined as 20–40 years old, with the main task of early adulthood being to establish intimate relationships. The middle-aged adults were defined as 40 to 65 years old, characterizing this period as a transition from early to late life stages. The MOEL 2017 report introduces a new paradigm for Koreans aged 50 to 69, termed the “New Middle-aged”. As Korea enters an aging society with increasing life expectancy, the government recognizes the need to revisit the definition of middle-aged. This conceptual shift necessitates a reevaluation of the traditional definition to reflect broader social changes. Based on these sources, this study defines young adults as 20 to 39 years old, utilizing Erikson’s criterion, and middle-aged adults as 40 to 69 years old, incorporating the MOEL’s expanded definition. Also, inspired by the concepts of ‘Digital Immigrants’ and ‘Digital Natives’ [30], this study will classify individuals into two groups: young adults (20 to 39 years old) and the middle-aged adults (40 to 69 years old).

The focus of this study will be to analyze which group utilizes digital devices more extensively and to identify the confidence of that age group. Based on this analysis, health management strategies utilizing digital devices will be proposed accordingly. This study is based on a survey on ‘Digital Health Accessibility and Personal Competency Factors’ conducted in 2021 by the Korea Institute for Health and Social Affairs (KIHASA).

## 2. Materials and Methods

### 2.1. Study Subjects and Data Collection

We utilized a unique panel survey dataset from the study titled “A Study on the Personal Capacity Building Model for Improving Access to Digital Health”, conducted between 16 December 2021 and 31 December 2021.

Data collection was conducted as part of the Korea Welfare Panel Survey, managed by the research company. The sampling frame comprised 1000 male and female individuals aged 20 to 69 from across the country. To ensure the representativeness of the survey participants, a ‘regional allocation’ was conducted for sample extraction by gender and age group based on the 17 cities and provinces. The survey was administered through a computer-based web interview utilizing a structured questionnaire.

The gender ratio of the surveyed population was 51.1% male and 48.9% female. The age distribution was as follows: 17.9% were in their 20s (20 to 29 years old), 18.0% were in their 30s (30 to 39 years old), 21.9% were in their 40s (40 to 49 years old), 23.3% were in their 50s (50 to 59 years old), and 18.9% were in their 60s (60 to 69 years old). For analysis, the population was divided into two groups: young adults in their 20s and 30s, referred to as Group 1, and middle-aged adults in their 40s, 50s, and 60s, referred to as Group 2.

### 2.2. Ethical Considerations

The online survey received approval from the Institutional Review Board (IRB) (Approval No. 2021-113) of the principal investigator’s institution, KIHASA, prior to data collection. Participation in the survey was voluntary. The research results derived from this survey can be made public after undergoing the personal data de-identification process in accordance with the law on the provision and utilization of public data. However, it has been previously stated that information that can identify individuals will be thoroughly protected.

### 2.3. Survey Methods

Among the sociodemographic variables, only age (20 to 69 years old) was examined.

The survey questionnaire included the following question regarding the use of digital health management tools: Q1 “Do you manage your health using wearable devices, mobile apps, or digital (non-face-to-face) methods? Please select option that you are currently utilizing”. The response options included nine categories: (1) wearable devices, (2) mobile apps, (3) video conferencing systems, (4) online videos, (5) telephone consultations, (6) video consultations, (7) other, (8) body composition analysis, and (9) none. For analytical purposes, responses in the “none” category were excluded.

Wearable devices used for health management includ pedometers, smart bands, smartwatches, and sneaker attachment measuring instruments. Examples of mobile applications mentioned were Samsung Health, LG Health, TOSS, Cash-walk, Nike Run, NOOM, Walk-On, Apple Health, OK-cashback, CashSlide, and AIA Vitality.

Regarding the use of digital devices/systems and confidence in gathering information, the survey included the following questions:
Q 2-1“I am well aware of how to use digital devices/systems”.Q 2-2“I am proficient in using the menus and features of digital devices/systems”.Q 2-3“I am confident in gathering information using digital devices/systems”.

The design of the above questionnaires was adapted from the survey of Choi 2020 [38]. Responses were recorded using a five-point Likert scale (1 = strongly disagree, 5 = strongly agree). In social science research, Likert-type items are commonly used for response formats, and a five-point scale is recommended for unipolar items [39,40]. In this study, a five-point Likert scale ranging from ‘strongly disagree’ to ‘strongly agree’ was employed.

Additionally, to assess confidence in using digital devices for health management, the following six questions were asked:
Q 3-1“I do not find it difficult to manage my health using digital devices/systems”.Q 3-2“I am confident in managing my health using digital devices/systems”.Q 3-3“I create my own plans to manage my health using digital devices/systems”.Q 3-4“I believe I can develop good health habits by utilizing digital devices/systems”.Q 3-5“I can consistently and repeatedly use digital devices/systems for health management”.Q 3-6“I can evaluate my health management results by utilizing digital devices/systems”.

The design of the aforementioned questionnaires was based on the work of Choi 2020 [38] and Van Der Vaart 2017 [41]. Van Der Vaart’s ‘Digital Health Literacy Instrument (DHLI)’ is a questionnaire that has been validated for reliability and validity through several studies and is already being effectively utilized by health organizations and institutions [42,43,44].

Responses were similarly recorded on a five-point Likert scale (1 = strongly disagree, 5 = strongly agree).

### 2.4. Statistical Analysis

Data were analyzed using IBM SPSS version 22.0 for Windows (IBM Corporation, Armonk, NY, USA) [45]. Table 1 indicates the number of people who utilize the digital device for health management in this survey. Table 2 presents the cross-tabulation analysis of utilization rates across each group. To assess the independence between young adults and middle-aged adults in this survey, a Chi-square test was conducted, as this statistical method is commonly used for analyzing relationships between nominal variables [46]. The chi-square value (χ^2^) was considered statistically significant at *p* < 0.05.

Independent samples *t*-tests were applied to data in Table 3 and Table 4 to determine statistically significant differences, with results highlighted in bold for *p* < 0.05. The primary outcome measures were derived from questions 2-1 through 2-3 and 3-1 through 3-6. Responses of “disagree” and “strongly disagree” were combined to indicate respondents’ hesitancy, while “agree” and “strongly agree” were combined to indicate confidence. Considering the nature of Likert-scale data, *t*-tests were utilized without concern for significant differences in power or error rates [47].

## 3. Results

### 3.1. Demography

There were 179 participants in the 20s age group, 180 participants in the 30s age group, 219 participants in the 40s age group, 233 participants in the 50s age group, and 189 participants in the 60s age group. Consequently, young-adults group comprised 359 individuals, while the middle-aged adults group comprised 641 individuals.

### 3.2. Usage of Digital Health Management

Upon examining both groups, 78.3% of individuals were engaged in digital health management. Excluding the 21.7% who were not, the analysis was conducted on the remaining 78.3%. Of the total participants, 217 were not utilizing digital devices for healthcare; therefore, the analysis was conducted on the remaining 783 respondents. As this question allowed for multiple -response, the focus was placed on the percentage rather than the absolute N value (Table 1).

Upon comparing the groups, the middle-aged adults group exhibited higher frequencies across all items compared to the young-adults group. This finding suggests that the middle-aged adults engage in digital health management more frequently than young-adults.

A cross-analysis was conducted to examine the differences between the groups. The results revealed a significant difference in the use of digital devices or systems for health management between the groups, with χ^2^ = 21.157 and *p* = 0.007. The analysis indicates that the likelihood of using digital devices or systems for health management increases with age (Table 2).

### 3.3. Confidence in Utilizing Digital Devices

An independent samples *t*-test was conducted to examine the difference in confidence in utilizing digital devices between the two groups. The group statistics are as follows: the young-adults group (N = 359) and the middle-aged adults group (N = 641) (Table 3).

According to the results presented in Table 3, the *t*-value for the first question, “I am well aware of how to use digital devices/systems”, is 6.417 with a significance level (*p*) of 0.000. For the second question, “I am proficient in using the menus and features of digital devices/systems”, the *t*-value is 6.748 with a significance level (*p*) of 0.000. The *t*-value for the final question, “I am confident in gathering information using digital devices/systems”, is 6.107 with a significance level (*p*) of 0.000. Consequently, the alternative hypothesis that “there is a difference in confidence in utilizing digital devices according to group” was accepted. For all three questions, the young-adults group demonstrated relatively higher confidence.

### 3.4. Confidence in Utilizing Digital Devices for Healthcare Management

An independent samples *t*-test was conducted to examine the difference in confidence in managing health using digital devices between the two groups. The group statistics are as follows: the young-adults group (N = 359) and the middle-aged adults group (N = 641) (Table 4).

Table 4 presents the differences in confidence between the groups in managing health using digital devices. The *t*-value for the first question, “I do not find it difficult to manage my health using digital devices/systems”, is 7.350 with a significance level (*p*) of 0.000. For the second question, “I am confident in managing my health using digital devices/systems”, the *t*-value is 6.258 with a significance level (*p*) of 0.000. The third question, “I create my own plans to manage my health using digital devices/systems”, has a *t*-value of 2.570 with a significance level (*p*) of 0.010. The fourth question, “I believe I can develop good health habits by utilizing digital devices/systems”, shows a t-value of 2.192 with a significance level (*p*) of 0.029. The fifth question, “I can consistently and repeatedly use digital devices/systems for health management”, has a *t*-value of 3.017 with a significance level (*p*) of 0.003. Lastly, the sixth question, “I can evaluate my health management results by utilizing digital devices/systems”, has a *t*-value of 4.215 with a significance level (*p*) of 0.000. Consequently, the alternative hypothesis that “there is a difference in confidence in health management using digital devices according to group” was accepted. The confidence levels for all six questions were significantly higher in the young-adults group compared to the middle-aged adults group, with all values being statistically significant.

## 4. Discussion

According to Jones et al., 2010, students aged 25 years and under, particularly those based in universities, were more confident in their skills related to ICT tasks. The survey also revealed that students are active users of technology and generally utilize it beyond what is required [48]. However, despite the high confidence and frequent use of digital devices among young adults, our survey results showed that the percentage of young adults actively using digital devices for healthcare was lower than that of middle-aged adults. This discrepancy can be interpreted in several ways. Primarily, young adults tend to use digital devices for purposes other than healthcare, such as social media and entertainment. Numerous studies suggest that young adults have an overwhelmingly positive view of the role of digital technologies in their daily lives, often regarding them as central resources for entertainment, information and communication [49,50,51,52]. This suggests that young adults may approach healthcare differently, opting not to rely on digital devices for health-related activities.

According to a study, young adults are increasingly concerned about the negative health effects associated with excessive digital device use, with 86% reporting that their inability to disconnect from digital devices outside of working hours adversely affects their well-being [53]. Prolonged use of digital devices has been associated with negative health outcomes, such as poor posture and impaired respiratory function [54,55]. Consequently, young adults have adopted practices like “digital detox” to manage their health, which may explain the lower utilization of digital devices for healthcare. Digital detox refers to intentionally taking breaks from digital device use to mitigate the risk of addiction [56]. Many young adults now engage in alternative activities, such as physical exercise, reading, and spending time outdoors, as part of this effort [53]. Studies have shown that digital detox can improve sleep quality, reduce stress, and enhance perceived health [57]. Thus, the health of the digital native (DN) generation may deteriorate due to the overuse of digital devices.

On the other hand, the middle-aged adults group exhibited lower confidence in using digital devices compared to the young-adults group, yet they reported higher utilization of digital devices for healthcare. There are several possible reasons for this.

First, middle-aged adults tend to feel a greater need for healthcare as they experience more physical changes and a higher likelihood of health problems. For instance, higher cardiorespiratory fitness in middle age is closely linked to reduced medical costs over time, regardless of cardiovascular risk factors [58]. This highlights the importance of exercise for maintaining quality of life in old age. Moreover, the prevalence of multimorbidity—defined as the coexistence of multiple chronic conditions—tends to increase with age, affecting approximately half of middle-aged adults and over 80% of those aged 75 and older [59,60]. However, engaging in muscle-strengthening activities has been associated with a 26% reduction in the likelihood of developing multimorbidity [61]. Given the broad benefits of physical activity on quality of life [62], also medical costs, middle-aged adults are more inclined to actively manage their health.

Second, with the advancement of digital technologies, middle-aged adults have gradually become more familiar with digital devices, enabling them to effectively use these tools for healthcare purposes. Ransdell et al. 2011 found that although older individuals reported lower confidence in using technology, they applied what they had learned more effectively than younger individuals. While older boomers did not grow up in the digital era, they are increasingly becoming proficient in online environments, particularly as students. Middle-aged adults, despite their relatively lower digital proficiency compared to young adults, may compensate through their extensive work and social experiences [63]. This allows them to use digital devices as effective healthcare tools.

Lastly, a report by Accenture, 2019 indicated that middle-aged and older adults are highly motivated to use digital health devices and are quickly adapting to them. The survey found that older adults displayed more favorable attitudes toward digital health devices than young adults. The use of health apps among the elderly increased five times from 2014 to 2018 (from 2.9% to 15.5%), and 95% of respondents indicated that they would actively share health data from apps or wearable devices with medical professionals [64].

The findings of this study suggest that healthcare strategies utilizing digital devices should be tailored to different age groups. The implementation of digital healthcare strategies for citizens should be conducted at the national level. Drawing from the plan proposed by the Ministry of Science and ICT of South Korea in 2022, which was developed in collaboration with relevant ministries for the effective utilization of intelligent information services, several e-Health strategies are presented [65]. While young adults demonstrate proficiency and confidence in using digital devices, they are also more susceptible to addiction. Therefore, it is crucial to develop digital healthcare strategies that incorporate elements of digital detox. For instance, this study proposes the use of digital technology to suggest lifestyle records such as diet, exercise, and sleep based on artificial intelligence, while simultaneously conducting research on technologies for analyzing and predicting factors of digital addiction. This approach can enhance the diagnostic scale for digital addiction and strengthen basic research to prevent it. Additionally, citizen participation projects are proposed in collaboration with influencers, national agencies, and companies on platforms frequently used by the young-adults, such as Instagram and YouTube. To support them in dedicating time to health management, citizen participation projects will be like digital healthcare strategies that incorporate elements of digital detox. As mentioned, young adults may be more susceptible to digital addiction and are more likely to engage in problematic digital device use due to their higher sensitivity to immediate rewards compared to older adults [66]. Conversely, middle-aged adults are already effective in managing their health using digital devices, but their confidence in using these tools is lower. Therefore, national policies that provide guidance on how to manage health using digital devices could further improve the health outcomes of middle-aged adults. For instance, a collaboration between the state, private sector, and medical community could facilitate telemedicine counseling and health management through the distribution of low-cost wearable devices such as smartwatches to middle-aged adults. This approach could enable the collection of health data and simplification of healthcare for this demographic. Additionally, providing education on technologies such as virtual reality and augmented reality at public health centers could support convenient tele-home training for middle-aged adults, conducted by physiotherapists.

### 4.1. Study Limitations

Several limitations should be noted. First, as this study is based on survey data, it relies on respondents’ subjective evaluations, and a causal relationship between digital device use and specific healthcare activities could not be definitively established. Furthermore, the online nature of the survey may have excluded individuals with limited digital access or skills, potentially biasing the sample towards those with greater digital literacy. Future studies should aim to collect more quantitative data through face-to-face interviews and explore direct relationships between digital device usage patterns and health outcomes across various ages, not the age groups. Additionally, the study’s age categorization may limit the generalizability of the findings, as different age groupings can provide various outcomes and affect the interpretation of behavioral trends. Therefore, future research should employ a more diverse age categorization and focus on the impact of digital familiarity across different generations. Moreover, the questionnaire from Choi 2020 [38] has not been extensively validated, suggesting that future surveys could benefit from utilizing additional reliable tools. Lastly, as the findings of this study were based on South Korean citizens, generalization to other countries may be inappropriate.

### 4.2. Study Strengths

This study provides important insights as the first attempt to compare the use of digital devices and healthcare behaviors between young and middle-aged adults. The findings demonstrate why these groups should approach digital healthcare methods differently and contribute to understanding the increased interest of middle-aged adults in digital healthcare.

## 5. Conclusions

To the best of our knowledge, this study investigated the differences in confidence regarding the use of digital devices and digital devices in healthcare between young and middle-aged adults. The findings indicate that while young adults exhibit high confidence in using digital devices, their utilization of these devices for healthcare purposes is relatively low. In contrast, middle-aged adults demonstrate lower confidence in using digital devices compared to young adults, yet they are more active in employing digital devices for healthcare management.

These results offer significant implications for the development of healthcare programs and policymaking [67]. For young adults, it is recommended to emphasize the importance of utilizing digital devices in healthcare practices, while ensuring caution to avoid over-reliance or addiction to such technologies. For middle-aged adults, it is necessary to develop customized programs that enhance the effective utilization of existing digital healthcare tools.

The clinical implications of digital healthcare research, tailored to different age groups, can be summarized as follows: Firstly, there is significant potential for the advancement of personalized medicine. By leveraging big data and artificial intelligence (AI), it becomes possible to develop precise diagnostic tools and personalized treatment plans that consider individual patient characteristics. AI-based clinical decision support systems can enhance diagnostic accuracy and treatment efficacy.

Secondly, healthcare accessibility can be greatly improved. Telemedicine enables medical consultations without situational or geographical constraints, while mobile healthcare applications support self-management of health. This could potentially lead to a long-term reduction in national healthcare response costs.

Thirdly, real-time health monitoring through wearable devices facilitates early diagnosis and disease prediction. This not only enhances preventive care but also increases the efficiency of clinical trials.

Lastly, and perhaps most significantly, these advancements collectively contribute to an overall improvement in the quality of healthcare. The integration of digital technologies in healthcare is expected to make substantial contributions to public health outcomes in the future and lead to improved medical decision-making.

In conclusion, digital healthcare research, when tailored to age-specific characteristics, presents numerous clinical implications that have the potential to revolutionize healthcare delivery, improve patient outcomes, and enhance overall public health.

## Figures and Tables

**Table 1:** Case Processing Summary.

Effective Value		Missing Value		Total Value	
**N**	**%**	**N**	**%**	**N**	**%**
783	78.3%	217	21.7%	1000	100.0%

Responses in the “none” category were excluded. N refers to the number of respondents. ‘Effective Value’ means the values used in the actual statistics, and ‘Missing Value’ refers to the values where non-respondents are treated as missing data.

**Table 2:** Cross Tabulation of Utilization Rates According to Group.

			Utilization of Digital Methods for Health Management and the Tools Employed								Total	χ^2^	*p*-Value
			Wearable Devices	Mobile Apps	Video Conferencing System	Online Video	Telephone Consultation	Video Consultation	etc.	Body Composition Analysis			
Group	1	N	186	216	8	44	14	5	5	1	290	**21.157**	**0.007**
		%	23.8%	27.6%	1.0%	5.6%	1.8%	0.6%	0.6%	0.1%	37.0%		
	2	N	274	342	26	105	43	16	6	2	493		
		%	35.0%	43.7%	3.3%	13.4%	5.5%	2.0%	0.8%	0.3%	63.0%		
Total		N	460	558	34	149	57	21	11	3	783		
		%	58.7%	71.3%	4.3%	19.0%	7.3%	2.7%	1.4%	0.4%	100.0%		

Table 2 was analyzed based on the values of ‘Effective Value’ from Table 1. Group 1: the young-adults group in their 20s and 30s; Group 2: the middle-aged adults group in their 40s, 50s, and 60s; N: the number of respondents for each item; the percentage: the proportion of respondents in each group; Obtained by complex chi-square test. Bold values denote statistical significance at *p* < 0.05.

**Table 3:** Statistics on Confidence in Utilizing Digital Devices for Each Group and Independent Samples Test on Questionnaires.

	Group	N	Mean	Std. Deviation	Levene’s Test		*t*-Test for Equality of Means						
					F	Sig.	*t*	df	Sig (2-Tailed)	Mean Difference	Std. Error Difference	95% CI	
												Lower	Upper
Q 2-1	1	359	3.91	0.860	4.888	0.27	6.417	719.419	**0.000**	0.359	0.056	0.249	0.469
	2	641	3.55	0.830									
Q 2-2	1	359	3.86	0.891	1.652	0.199	6.748	998	**0.000**	0.389	0.058	0.276	0.502
	2	641	3.47	0.864									
Q 2-3	1	359	3.79	0.903	0.029	0.865	6.107	998	**0.000**	0.356	0.058	0.242	0.471
	2	641	3.43	0.874									

Group 1: the young-adults group in their 20s and 30s; Group 2: the middle-aged adults group in their 40s, 50s, and 60s; N: the number of respondents for each item; the percentage: the proportion of respondents in each group; Q 2-1: “I am well aware of how to use digital devices/systems”; Q 2-2: “I am proficient in using the menus and features of digital devices/systems”; Q 2-3: “I am confident in gathering information using digital devices/systems”; Bold values denotes statistical significance at *p* < 0.05.

**Table 4:** Statistics on Confidence in Utilizing Digital Devices for Healthcare Management for Each Group and Independent Samples Test on Questionnaires.

	Group	N	Mean	Std. Deviation	Levene’s Test		*t*-Test for Equality of Means						
					F	Sig.	*t*	df	Sig (2-Tailed)	Mean Difference	Std. Error Difference	95% CI	
												Lower	Upper
Q3-1	1	359	3.91	0.872	7.623	0.006	7.350	730.870	**0.000**	0.420	0.057	0.308	0.532
	2	641	3.49	0.857									
Q3-2	1	359	3.68	0.918	3.490	0.062	6.258	998	**0.000**	0.366	0.058	0.251	0.480
	2	641	3.32	0.868									
Q3-3	1	359	3.39	1.079	9.986	0.002	2.570	678.615	**0.010**	0.177	0.069	0.042	0.311
	2	641	3.21	0.972									
Q3-4	1	359	3.69	0.904	5.758	0.017	2.192	655.005	**0.029**	0.125	0.057	0.013	0.236
	2	641	3.57	0.780									
Q3-5	1	359	3.79	0.887	0.044	0.835	3.017	998	**0.003**	0.167	0.055	0.059	0.276
	2	641	3.62	0.815									
Q3-6	1	359	3.68	0.906	2.505	0.114	4.215	998	**0.000**	0.235	0.056	0.125	0.344
	2	641	3.45	0.809									

Group 1: the young-adults group in their 20s and 30s; Group 2: the middle-aged adults group in their 40s, 50s, and 60s; N: the number of respondents for each item; the percentage: the proportion of respondents in each group; Q 3-1: “I do not find it difficult to manage my health using digital devices/systems”; Q 3-2: “I am confident in managing my health using digital devices/systems”; Q 3-3: “I create my own plans to manage my health using digital devices/systems”; Q 3-4: “I believe I can develop good health habits by utilizing digital devices/systems”; Q 3-5: “I can consistently and repeatedly use digital devices/systems for health management”; Q 3-6: “I can evaluate my health management results by utilizing digital devices/systems”; Bold values denotes statistical significance at *p* < 0.05.

## Data Availability

Data are available from Korea Institute for Health and Social Affairs (KIHASA) for all interested researchers can request to use.
